# Functional dissection of *Drosophila melanogaster* SUUR protein influence on H3K27me3 profile

**DOI:** 10.1186/s13072-017-0163-z

**Published:** 2017-12-01

**Authors:** Olga V. Posukh, Daniil A. Maksimov, Petr P. Laktionov, Dmitry E. Koryakov, Stepan N. Belyakin

**Affiliations:** 1 Genomics Lab, Institute of Molecular and Cellular Biology SB RAS, Lavrentyev ave. 8/2, Novosibirsk, Russia 630090; 2 Novosibirsk State University, Pirogov str. 2, Novosibirsk, Russia

**Keywords:** Heterochromatin, Replication, Polycomb, H3K27me3, Epigenetic inheritance, Drosophila

## Abstract

**Background:**

In eukaryotes, heterochromatin replicates late in S phase of the cell cycle and contains specific covalent modifications of histones. *SuUR* mutation found in Drosophila makes heterochromatin replicate earlier than in wild type and reduces the level of repressive histone modifications. SUUR protein was shown to be associated with moving replication forks, apparently through the interaction with PCNA. The biological process underlying the effects of SUUR on replication and composition of heterochromatin remains unknown.

**Results:**

Here we performed a functional dissection of SUUR protein effects on H3K27me3 level. Using hidden Markow model-based algorithm we revealed *SuUR*-sensitive chromosomal regions that demonstrated unusual characteristics: They do not contain Polycomb and require SUUR function to sustain H3K27me3 level. We tested the role of SUUR protein in the mechanisms that could affect H3K27me3 histone levels in these regions. We found that SUUR does not affect the initial H3K27me3 pattern formation in embryogenesis or Polycomb distribution in the chromosomes. We also ruled out the possible effect of SUUR on histone genes expression and its involvement in DSB repair.

**Conclusions:**

Obtained results support the idea that SUUR protein contributes to the heterochromatin maintenance during the chromosome replication. A model that explains major SUUR-associated phenotypes is proposed.

**Electronic supplementary material:**

The online version of this article (10.1186/s13072-017-0163-z) contains supplementary material, which is available to authorized users.

## Background

In higher eukaryotes, transcriptionally active and silent regions of the genome are known to replicate asynchronously. Heterochromatic regions complete replication late in S phase when the rest of the chromosome has already been copied [1–3]. This replication pattern is basically determined by the density of replication initiation sites (origins) and their firing schedule: Euchromatin is enriched with early firing origins, while in heterochromatin replication origins are mostly depleted [4, 5]. The closer the two active origins are to each other, the faster the region between them gets replicated. Thus, the latest to replicate would be the regions located between the most distant neighboring late firing origins.

As in other metazoans, heterochromatic state in Drosophila is established by two major repressive pathways: HP1-dependent [6] and Polycomb-dependent [7]. These pathways result in the formation of repressed chromosomal domains marked by H3K9me2/3 and H3K27me3, respectively. Su(var)3–9 protein is responsible for di- and tri-methylation of H3K9 mainly at pericentric regions [8]. This mark is recognized by the chromodomain of HP1 protein [9] that, together with other heterochromatic factors, completes the formation of specific chromatin state in pericentric regions. The repressive pathway that silences developmentally regulated genes throughout the genome is based on the interplay of Polycomb-repressive complex 2 (PRC2 encompassing E(Z) and Su(z)12 proteins) and PRC1 complex, which contains a chromodomain protein Polycomb (Pc). PRC2 places methylation mark on H3K27, and PRC1 binds this mark and causes gene repression [7]. Both heterochromatin types resulting from these repressive pathways complete replication late in S phase [5]. However, other than the lack of origins, little is known about the factors that make heterochromatin replicate late.

In salivary gland cells of Drosophila larvae, polytene chromosomes are formed when, after several sequential endocycles lacking mitosis and cell division, 500–1000 DNA strands stay tightly bound together by cohesin molecules [10]. In polytene chromosomes, some late replicating regions fail to complete replication and contain fewer DNA strands [11–14]. The phenomenon of under-replication is caused by truncated S phase in endocycling salivary gland cells: Replication forks in the latest replicating regions of polytene chromosomes fail to converge, thus resulting in accumulation of DNA double-stranded breaks (DSBs) at these sites [15–18].

Phenomenally, under-replicated regions complete replication earlier and become fully polytenized in *Suppressor of Under-replication* (*SuUR*) mutants [19, 20]. The effect of *SuUR* mutation on polytenization is reached without altering the origin distribution [13], suggesting that exact same regions of chromosomes replicate more efficiently in *SuUR* mutants than in wild type, where SUUR protein is functional. Extra copies of *SuUR* gene lead to increased under-replication in polytene chromosomes [20].These data argue that the normal function of SUUR protein in the cell is to actively impede replication of heterochromatin; however, the biological function of this process remains unclear [13, 15].

Recent study revealed that *SuUR* mutation affects the levels of H3K27me3 and H3K9me3 marks that are depleted in the pericentric regions of *SuUR* mutants [21]. Furthermore, under-replicated regions that become fully polytenized in *SuUR* mutants lose H3K27me3 mark [13]. Nevertheless, SUUR protein fails to affect gene expression [13, 22]. Thus, *SuUR* mutation results in two major effects on polytene chromosomes: Chromosomal regions that are under-replicated in wild type become fully polytenized [19]; these regions lose most of their H3K27me3 histones [13].

SUUR protein interacts with replication complex indicating that it is directly involved in replication process [15, 23]. A recent study demonstrated a link between SUUR and linker histone H1 [24]. Importantly, H1 knockdown leads to increased polytenization in the normally under-replicated regions and the presence of H1 seems to be essential for SUUR stability. H1 demonstrated a dynamic distribution in polytene chromosomes through the S phase, although not in the same way as SUUR protein [23, 24]. Discovered interactions of SUUR protein are insufficient to decipher the molecular mechanism of its action at the replication fork, and the effect of *SuUR* mutation on repressive histone modifications remains poorly studied.

In this work, we explore the effect of SUUR on H3K27me3 level using polytene chromosomes as a model system. We performed a comprehensive analysis of chromosomal regions that are sensitive to *SuUR* mutation either in the context of polytenization or H3K27me3 levels. We showed that the formation of H3K27me3 domains in early embryos is unaffected by *SuUR* mutation. Our results support the idea that SUUR is a part of the mechanism that specifically re-establishes repressive chromatin during the chromosome replication [25]. As these mechanisms are still insufficiently studied, our work provides an essential example that may help uncover the biological aspects of epigenetic inheritance.

## Results

The first step to deciphering the mechanism involving SUUR protein was to establish causal relationship between the H3K27 methylation and under-replication phenomenon. Indeed, locally elevated polytenization level could result in the decrease in H3K27me3 ChIP/input signal in *SuUR* mutants observed in previous studies [13, 21]. For example, in wild type, H3K27 methylation in these regions could be a response to under-replication and double-stranded DNA breaks [26, 27], which disappear in *SuUR* mutants [15, 18]. Alternatively, under-replication could be a consequence of the local repressed chromatin state that impedes replication in SUUR-dependent manner.

**Fig. 1:**
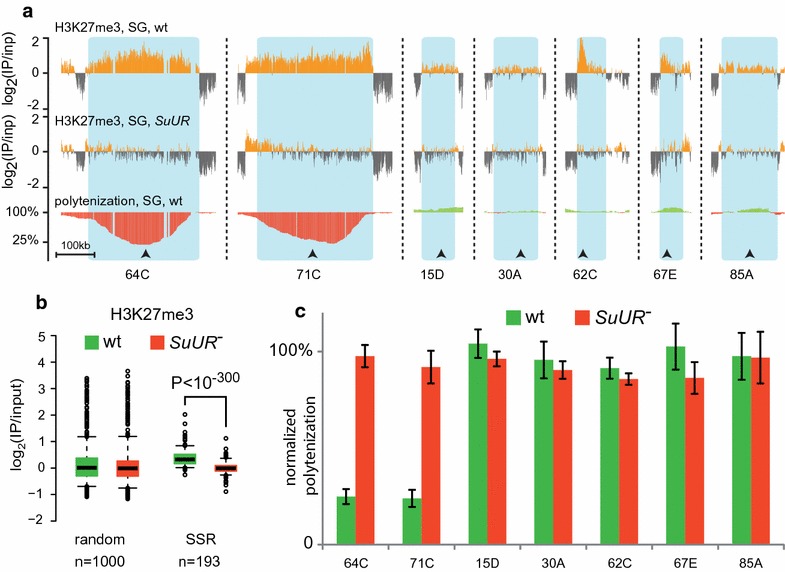
*SuUR* mutation affects H3K27me3 level independently from local under-replication. **a** Examples of chromosomal regions that display decrease in H3K27me3 levels upon *SuUR* mutation, but are fully polytenized in wild type. Compared to under-replicated regions 71C and 64C. Polytenization data were taken from [13]. Arrowheads show the positions of qPCR primers that were used to validate polytenization levels in these regions. Blue shading demarks SSRs identified with HMM. **b** SSRs identified with HMM display systematic decrease in H3K27me3 levels upon *SuUR* mutation. Compared to 1000 random chromosomal regions. **c** qPCR analysis of the polytenization levels in SSRs, shown in (**a**)

If the elevated polytenization level is primary to the H3K27me3 loss in *SuUR* mutants, this effect would be restricted only to the regions that are under-replicated in wild type and would never be found in the regions that are fully polytenized. However, comparing the H3K27me3 profiles to the polytenization levels published in [13], we found that the loss of H3K27me3 upon *SuUR* mutation is not restricted to the under-replicated sites, but also appears in many regions that are fully polytenized (exemplified in Fig. 1a).

To perform a systematic analysis, we applied HMM-based approach (Materials and Methods) to the published ChIP-chip profiles [13] and detected genomic regions where H3K27me3 levels were sensitive to *SuUR* mutation (*SuUR*-sensitive regions, SSRs) regardless of their polytenization levels. H3K27me3 ChIP-chip data were quantile normalized prior to the analysis. This approach identified 193 chromosomal areas (each spanning over 30 kb; average length 214 kb; average H3K27me3 normalized ChIP/input signal in wild type 0.41, in *SuUR* mutants − 0.06, paired *t* test *P* = 2.3 × 10^−54^; Additional file 1: Table S1) that manifest decreased H3K27me3 levels in *SuUR* mutants as detected by the HMM algorithm (Fig. 1b).

It has to be mentioned that the antibodies (Abcam, #6002) that were used to demonstrate the effect of *SuUR* mutation on H3K27 methylation level [13] predominantly recognize H3K27me3, but were also reported to cross-react with H3K27me2 (about 12% cross-reactivity as determined by ELISA, see product information on the Abcam website). Thus, it was possible that ChIP profiles obtained with these antibodies represent both modifications. To address this issue, we performed the independent ChIP-seq profiling of H3K27me3 (with Cell Signaling Technology #9733 antibodies) and H3K27me2 (with Millipore #07-452 antibodies) in salivary glands of *SuUR* mutants and in wild type control and compared them to the published ChIP-chip profiles obtained with Abcam #6002 antibodies. The result of this comparison summarized in the Additional file 2: Figure S1 indicates that the effect of *SuUR* mutation [13] is indeed directed on H3K27me3 and does not involve H3K27me2.

Then, we compared *SuUR*-sensitive regions detected by HMM to the previously published under-replication regions [12–14]. For this analysis, we combined data from these three studies and formed a list of 101 regions, which displayed under-replication in at least one of the three studies (Additional file 3: Table S2). As expected, 99 out of 193 SSRs overlapped with the list of previously reported under-replicated regions [12–14] (average length 232 kb; average H3K27me3 normalized ChIP/input signal in wild type 0.48, in *SuUR* mutants − 0.06, paired *t* test *P* = 1.1 × 10^−40^). However, 94 SSRs had no overlap with any under-replicated areas (average length 195 kb; average H3K27me3 normalized ChIP/input signal in wild type 0.23, in *SuUR* mutants − 0.06, paired *t* test *P* = 6.3 × 10^−22^). Figure 1a and Additional file 4: Figure S2 demonstrates several examples of such fully polytenized SSRs. Remarkably, SSRs that did not show under-replication in wild type demonstrated somewhat lower average H3K27me3 signals as compared with the under-replicated SSRs (0.23 vs. 0.48, see above), suggesting that the ability of SUUR to interfere with replication correlates with local H3K27me3 level.

We confirmed DNA polytenization levels in regions shown in Fig. 1a by qPCR. As seen in 71C and 64C regions, the most prominent under-replication occurs approximately in the center of the corresponding SSRs. We designed qPCR primers to target the middle part of each SSR shown in Fig. 1a. Polytenization was measured in wild type larval salivary glands and in *SuUR* mutants. *Actin 42A* gene was used for total DNA normalization, as this gene gets fully polytenized regardless of *SuUR* background [28, 29]. Figure 1c demonstrates that tested regions manifesting the loss of H3K27me3 upon *SuUR* mutation show no signs of under-replication in wild type. This whole-genome analysis with independently confirmed examples indicates that *SuUR* mutation affects H3K27me3 abundance in a more intricate way than modulation of polytenization level.

It may be suggested that SUUR protein is involved in the initial formation of H3K27me3 pattern in chromosomal regions that we identified as SSRs. As epigenetic patterns are established in early development, the effect of *SuUR* mutation on H3K27me3 pattern observed in salivary gland cells could already be displayed in early embryos. To address this question directly, we performed ChIP-seq analysis of H3K27me3 distribution in 0–4 h embryos of wild type strain and *SuUR* mutants. At this developmental stage, *SuUR* mRNA is highly abundant in wild type strains [30] and is uniformly distributed throughout the embryo [31–33]. Notably, as *SuUR* mutants are viable and fertile [19], mutant homozygous stock is maintained for almost 20 years ensuring that mutant embryos are free from functional *SuUR* gene product of either maternal or zygotic origin.

**Fig. 2:**
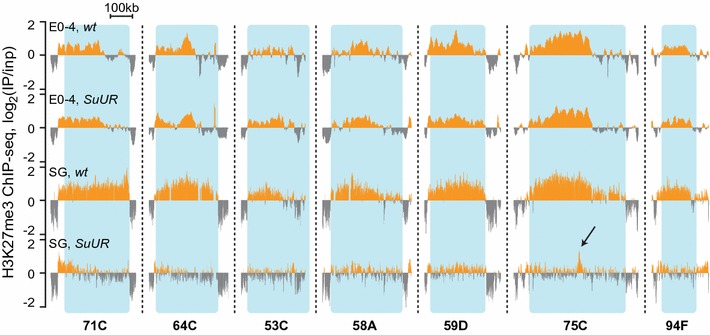
H3K27me3 profile is unaffected by *SuUR* mutation in early embryos. Examples of H3K27me3 profiles in some SSRs, in 0–4-h embryos and in salivary glands of wild type strain and *SuUR* mutants. No major changes occur upon *SuUR* mutation in embryos as compared to salivary glands. The arrow shows the position of Pc-binding site in 75C that is unchanged between wild type and *SuUR* mutants (see below)

Figure 2 demonstrates that in early embryonic development H3K27me3 ChIP-seq signal within SSRs displays no difference in *SuUR* and wild type. This observation indicates that SUUR is dispensable for the initial H3K27me3 pattern formation in early embryonic development.

**Fig. 3:**
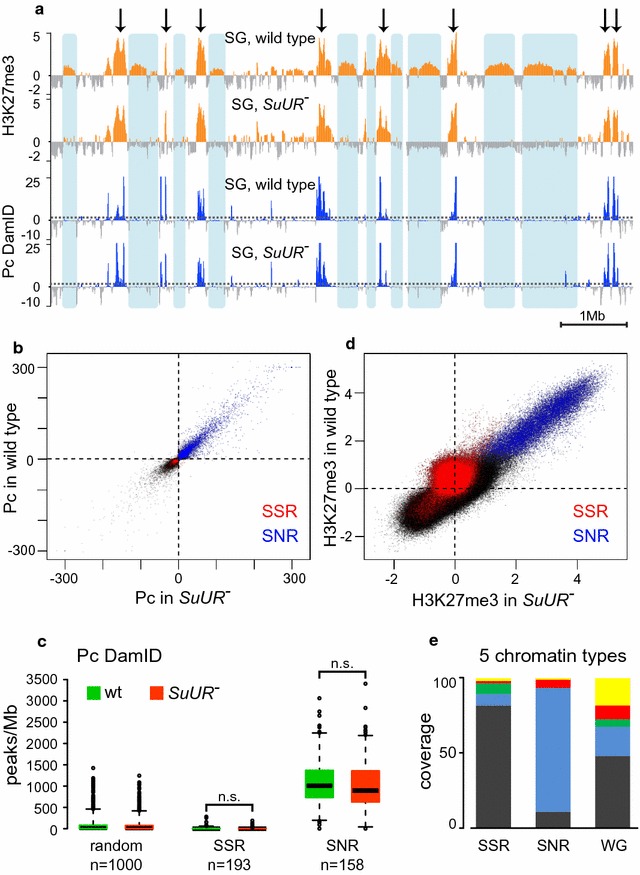
SSRs are devoid of Pc protein while SNRs represent PRC1 domains. **a** SSRs (shaded in blue) are devoid of Pc binding as exemplified by 10 Mb span of chromosome 2L. SNRs (see text) match the Pc-bound domains (arrows). H3K27me3 profiles are presented as quantile normalized log_2_(IP/inp) values. Pc DamID profiles are presented as log_10_(P) units, where P—significance level assessed with Fisher’s exact test. Dotted lines designate FDR = 0.5 levels for Pc enrichment. Blue shading shows positions of SSRs, arrows point at SNRs. **b** Scatter plot of Polycomb DamID data in salivary glands of wild type strain and in *SuUR* mutants. SNRs are shown in blue while SSRs—in red. Pc DamID signal is presented as log_10_(P) units, where P—significance level assessed with Fisher’s exact test. **c** Pc peak density in SSRs, SNRs and in 1000 random regions across the genome. *SuUR* mutation does not affect Pc distribution nor in SSRs nor in SNRs. **d** Scatter plot of quantile-normalized H3K27me3 ChIP-chip data in salivary glands of wild type strain and in *SuUR* mutants. SNRs are shown in blue while SSRs—in red. The scales are log_2_(IP/inp) values. **e** Genomic regions corresponding to SSRs and SNRs in Kc167 cells show characteristic distribution of chromatin types according to the classification of [5]

H3K27me3 histone modification is associated with Polycomb-mediated repression and is specifically bound by Pc protein [34]. *SuUR* mutation could disrupt PRCs binding in SSRs, which in turn could affect the H3K27me3 levels. Immunostaining of polytene chromosomes of *SuUR* mutant larvae has previously revealed no change in Pc-binding sites [20, 35]. However, that cytological study considered only the major Pc sites and did not report the information on the regions that we identified as SSRs. To address this question, we performed a whole-genome Polycomb DamID-seq mapping in salivary glands of *SuUR* mutants and wild type strain. Obtained data were analyzed as described previously [36, 37], for peak calling a 5% FDR threshold was used. Resulting profiles appeared to be very similar: Positions of most if not all Pc peaks were identical in *SuUR* and wild type polytene chromosomes (Fig. 3a, blue profiles). Pearson’s correlation value observed for the Pc profiles in two genotypes is 0.93, which is very close to the value found in the pairs of biological replicates within each genotype (0.98). In other words, *SuUR* mutation had nearly no effect on Pc binding in salivary gland polytene chromosomes. Any differences found in *SuUR* mutants were minor and essentially comparable to the experimental noise. Hence, we conclude that Polycomb profile is unaffected by *SuUR* mutation. Taken together, our H3K27me3 ChIP-seq and Pc DamID-seq data show that SUUR is not directly involved to the mechanisms of Polycomb-mediated repressed chromatin establishment.

Two distinct types of H3K27me3-enriched regions are clearly seen in salivary gland cells (Fig. 3a). One type is SSRs, all of which appeared to have rather moderate levels of H3K27me3 ChIP-seq signal in wild type and lose this mark upon *SuUR* mutation (Fig. 3a). The other type consists of the regions with high H3K27me3 ChIP-seq signal that, unlike SSRs, retain this mark in *SuUR* mutants (Fig. 3a, indicated with arrows). HMM approach identified 158 sites of high H3K27me3 abundance that were not sensitive to *SuUR* mutation (*SuUR*-non-sensitive regions, SNRs; Additional file 5: Table S3). These sites of high H3K27me3 abundance perfectly matched the Pc-enriched regions both in wild type and *SuUR* mutants (Fig. 3a), and Pc profile in these regions was independent from SUUR in our genome-wide analysis (Fig. 3b). Most SSRs appeared to be virtually devoid of Pc both in wild type and *SuUR* mutant genotypes (Fig. 3a–c), and moderately enriched with H3K27me3 in wild type (Figs. 1a, 3d).

To get a more detailed overview of SSRs and SNRs, we used the model that classifies chromatin of Drosophila Kc167-cultured Drosophila cells into five major types [5]. This model distinguishes three types of heterochromatin (color-coded as BLACK, BLUE and GREEN) and two types of active chromatin types (RED and YELLOW). We estimated the representation of these five chromatin types in the genomic spans of Kc167 cells corresponding to SSRs and SNRs (Fig. 3e). As expected, BLUE chromatin corresponding to repressed, PRC1 and PRC2-enriched chromatin [5] was highly represented within the limits of SNRs. In turn, genomic spans corresponding to SSRs in Kc167 cells were predominantly covered by BLACK chromatin (Fig. 3e). BLACK chromatin covers about a half of Kc167 chromosomes and is prevalent among the repressed chromatin types. Notably, as well as SSRs in polytene chromosomes, BLACK chromatin in Kc167 cells is not bound by Pc and shows a slightly elevated level of H3K27me3 [5].

**Fig. 4:**
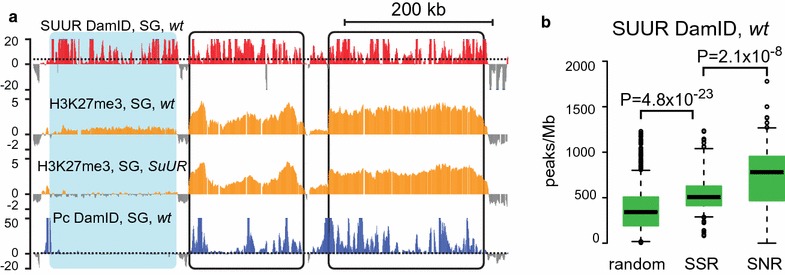
Both SSRs and SNRs contain SUUR protein. **a** DamID profiling detects SUUR protein in SSR (shaded in blue) and in SNRs (black frames). The 600-kb fragment of chromosome 3R is shown. H3K27me3 profiles are presented as quantile normalized log_2_(IP/inp) values. SUUR and Pc DamID profiles are presented as log_10_(P) units, where P—significance level assessed with Fisher’s exact test. Dotted lines designate FDR = 0.5 levels for Pc enrichment. **b** SUUR peak density in SSRs, SNRs and in 1000 random regions across the genome

Polycomb enrichment in SNRs suggests that the presence of PRCs accounts for the local restoration of H3K27 methylation in these regions, thus allowing H3K27me3 levels in these regions withstand the effect of *SuUR* mutation. On the other hand, it was possible that *SuUR* mutation had no effect on H3K27me3 levels in SNRs because these regions could be devoid of SUUR protein in polytene chromosomes. This possibility is unlikely, considering previous studies in salivary glands [20, 35] and cell culture [5, 38]. However, to ascertain that SUUR protein does not avoid SNRs in salivary glands, we performed a direct test. We applied DamID-seq method to build SUUR protein profile in salivary gland polytene chromosomes. Resulting profile (Additional file 6: Figure S3) was very similar to the profile previously obtained using microarrays in Kc167 cells [5] with some expectable cell type-specific differences [39]. Figure 4a illustrates the chromosomal region encompassing two SNRs and a neighboring SSR. As in other cases, both SNRs are enriched with Polycomb-binding sites and show no difference in H3K27me3 levels in wild type and *SuUR* mutants. Upper profile shows SUUR protein localization determined by DamID-seq [36]. As one can see, SUUR protein is present in both SSR and SNRs in comparable amounts.

To demonstrate how this trend translates to the whole-genome level, we measured the number of SUUR protein peaks per Mb in all SSRs and SNRs. This analysis revealed that in salivary glands SNRs demonstrate a strong presence of SUUR, even higher than in *SuUR*-sensitive regions (Fig. 4b). Nevertheless, H3K27me3 levels in SNRs were unaffected by *SuUR* mutation. Apparently, the presence of PRCs that re-introduce H3K27me3 mark is sufficient to overcome the effect of *SuUR* mutation. In contrast, SSRs devoid of Pc cannot rely on this compensatory mechanism and require SUUR function to sustain this repressive mark.

Next, we aimed to explore the role of histones in under-replication using mutation in the *mxc* gene (*multiple sex combs*). This mutation results in replication stress and homeotic phenotypes reminiscent of Polycomb group mutations. Mxc protein specifically regulates histone genes expression in cell cycle, but is not a part of the PRC complexes. The effects of *mxc* mutation are apparently mediated by the several fold over-production of H3 histone and are rescued by *His3* mRNA depletion [40].

**Fig. 5:**
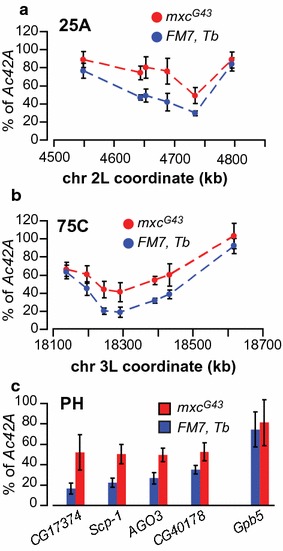
*mxc* ^*G43*^ mutants increase polytenization in under-replicated regions. **a**, **b** Polytenization profiles in two under-replicated regions 25A and 75C were built using qPCR in *mxc* ^*G43*^ mutants (red) and their wild type siblings (blue). **c** Polytenization levels of the tags from the pericentric heterochromatin were assessed with qPCR. Although located in pericentric heterochromatin, *Gbp5* gene is 100% polytenized in all genotypes and is shown for comparison. *mxc* ^*G43*^ mutation affects under-replication in both pericentric heterochromatin and in the late replicating regions scattered along chromosomal arms

To check if misregulation of histone genes in *mxc* mutants also affects under-replication, we measured DNA polytenization in salivary glands of viable hemizygous *mxc* ^*G43*^ male third instar larvae. *FM7, Tb* [41] balancer brothers were used as a control. We chose two under-replicated regions [12] from H3K27me3-marked heterochromatin (25A and 75C), which were characterized as SSRs. We designed qPCR primer pairs targeting genomic loci approximately every 50 kb within these regions to build detailed polytenization profiles (Fig. 5a, b). Additionally, we tested several under-replicated loci from pericentric heterochromatin (Fig. 5c). *mxc* ^*G43*^ mutants demonstrated substantial increase in polytenization levels in 25A and 75C regions as well as in the pericentric regions, which strongly supports the idea that under-replication is controlled at the histone level.

The effect of *mxc* mutation leaves a possibility that *SuUR* mutation could likewise affect under-replication through misregulation of histone genes. This possibility seemed quite plausible as the recent study [24] demonstrated that H1 depletion leads to the effects on polytenization similar to *mxc* mutation. To address this possibility, we measured the expression levels of histone genes in salivary glands of *SuUR* mutants using qPCR and compared them with the wild type strain. *SuUR* mutants showed no significant differences in expression levels of H1 and H3 histones (and other histones) compared with wild type (Additional file 7: Figure S4), indicating that SUUR does not affect under-replication through the histones synthesis regulation.

## Discussion

**Fig. 6:**
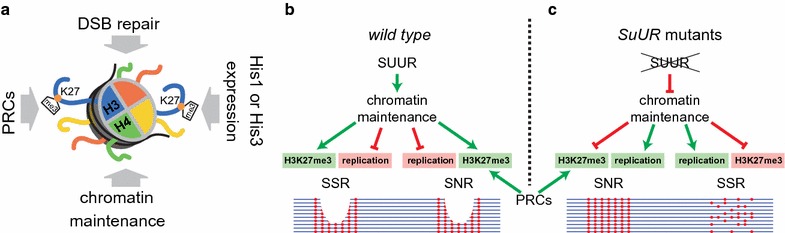
Scheme explaining the effects of SUUR on H3K27me3 maintenance and under-replication in polytene chromosomes. **a** Possible mechanisms that were checked in this study that could affect H3K27me3 in *SuUR* mutants. **b** In wild type, SUUR contributes to chromatin maintenance in a way that replication forks are delayed to allow the H3K27me3-enriched chromatin context to be re-established. This would result in under-replication in the most extended SSRs and SNRs. PRCs also act in SNRs and produce H3K27 methylation. **c** In *SuUR* mutants, SUUR-dependent chromatin maintenance is inactivated, so the replication is more efficient while H3K27me3 is lost in SSRs. In SNRs, H3K27me3 level is reconstituted by the resident PRCs. Below the schematic pictures of polytene chromosomes are shown. Red dots—H3K27me3 histones

This study continues our efforts to decipher the function of SUUR protein in Drosophila. *SuUR* mutation affects two processes in the repressed regions of polytene chromosomes—their polytenization and repressed histone modifications maintenance. In *SuUR* mutants, the replication in these regions becomes more efficient [13, 15, 19]; however, the levels of H3K27me3 and H3K9me3 decrease significantly [13, 21]. We performed a “differential diagnosis” for the effects of *SuUR* mutation on H3K27me3 level in polytene chromosomes. Successive conclusions allowed us to finally exclude SUUR involvement in certain mechanisms that, to this point, obscured the assessment of its function (Fig. 6a). We tested four potential explanations for the remarkable, but insufficiently studied, effects that SUUR has on chromosome replication and chromatin. Our study revealed that H3K27 methylation in SSRs of wild type chromosomes does not happen in response to DSB formation during under-replication as was shown in other model systems [26, 27]. Indeed, many regions that are 100% polytenized in wild type contain H3K27me3 that is sensitive to *SuUR* mutation. We also showed that *SuUR* mutation does not affect Polycomb DamID profile in salivary gland and is not involved in the initial placement of H3K27me3 mark early in embryogenesis. These results indicate that the effect of *SuUR* mutation on H3K27me3 level develops during the ontogenesis. Finally, we excluded the possibility of SUUR protein regulating the expression of histone genes.

In our previous work, we proposed a hypothesis that SUUR protein is involved in the maintenance of repressive histone modifications during replication in Drosophila [25]. We suggested that SUUR protein could function in the replication-coupled re-establishment of repressed histone modifications in polytene chromosomes. According to our model, SUUR impedes the progression of the replication complex through heterochromatin regions until the pattern of repressed histone marks is properly re-established on the newly synthesized DNA strands (or until the context for future chromatin maturation is properly formed). In the absence of this regulation, replication forks progress through heterochromatin regions more efficiently, but at the expense of the significant depletion of H3K27me3 and H3K9me3. This model combines all major effects of SUUR protein and provides a causal link between them. In this study, we performed necessary experiments to test this model in context of SUUR effect on H3K27me3.

New data obtained in this study add fascinating details to the well-known effects of SUUR protein. Our analysis of the published H3K27me3 profile in salivary gland chromosomes [13] revealed two distinct types of H3K27me3-containing regions—SSRs and SNRs—that differ in H3K27me3 levels, sensitivity to *SuUR* mutation and the presence of Pc. Intriguingly, the reduction in H3K27me3 levels upon *SuUR* mutation is observed only in regions that are moderately enriched with H3K27me3 and lack Pc, whereas highly enriched regions remain unaffected. Although, SUUR DamID signal in SNRs is even higher than in SSRs, which is consistent with early cytological studies [20, 35]. Thus, SUUR function is required to preserve H3K27me3 levels at the regions, which are devoid of Pc.

The majority of SSRs detected in this study overlap with the BLACK chromatin type (Fig. 3e) described in Kc167 cells [5]. Genomic regions corresponding to BLACK chromatin were recently shown to contain H3K27me2 in Sg4 cells [42]. Given the known cross-reactivity of the antibodies, which were used in ChIP experiments that detected *SuUR* effect on H3K27 methylation level [13], it could be suggested that SSRs mainly contain di-methylated H3K27 and *SuUR* mutation affects the level of this modification. This suggestion contradicts with the previous immunostaining results that showed no effect of *SuUR* mutation on H3K27me2 level [21]; however, the effect may be too subtle to be detected with the cytological methods. The present study proved that the previously observed effect of *SuUR* mutation [13] is specifically directed on tri-methylated H3K27; however, to further address the mechanism of SUUR action in chromatin it would be useful to study the effects of this protein considering a wider spectrum of histone modifications.

It is highly plausible that Pc-G proteins maintain H3K27 methylation at their target regions (Fig. 6b, c) by temporarily over-producing H3K27me3-marked histones prior to replication, as reported earlier [43]. Hence, the regions of high H3K27me3 enrichment resist the effect of *SuUR* mutation.

Although the presence of Polycomb protein in SNRs apparently compensates the effect of *SuUR* mutation on H3K27me3 level, it fails to neutralize the effect of SUUR protein on the replication in these regions. Indeed, the very first characterized under-replicated region (89DE) contains Bithorax complex, which is densely covered with Pc, but is still under-replicated in wild type and fully polytenized in *SuUR* mutants [12, 44]. Similar situation is observed in the Antennapedia complex [13, 14]. Both Bithorax complex and Antennapedia complex are as large as 200–300 kb, so it seems that under-replication normally occurs at H3K27me3-enriched regions that exceed a certain length and lack internal replication origins. This suggestion is in line with recently discovered negative correlation between the length of the under-replicated regions and their polytenization levels [14, 24]. Hence, the selectivity of *SuUR* mutation effect on H3K27me3 level turns out to be associated with the presence of Pc protein. However, the effect of *SuUR* mutation on under-replication apparently is largely dependent on the size of the repressed domain, rather than its overall level of H3K27me3. These conclusions are consistent with earlier cytological observations based on immunostaining [20]. Discovered SUUR protein effects on replication and chromatin in polytene chromosomes are schematically summarized in Fig. 6b, c.

The fact that SSRs do not bind Pc suggests two plausible mechanisms of how SUUR could maintain the level of H3K27me3 in these regions. On the one hand, SUUR could mediate the interaction between the replication complex and H3K27-specific methylase PRC2 and possibly other histone-modifying enzymes [45]. On the other hand, SUUR could regulate the incorporation of parental modified histones (or those over-produced by PRCs at their binding sites) into nascent chromatin of SSRs. Notably, a recent study suggests that linker histone H1 may be involved in this process [24]. Future studies will elucidate the exact mechanism of this process.

## Conclusions

Little is known about the specific maintenance mechanisms for repressive histone marks, but those few that are known to date appear to be very distinct. Recent reports indicate that during replication in mammalian cells parental H3K9me3 and H3K27me3 histone molecules are diluted twofold with new unmodified histones and then follows a lengthy (up to several hours) process of the re-establishment of histone methylation to original levels [46, 47]. A study in Drosophila revealed that over-production of H3K27me3 in early S phase at the Polycomb-binding sites secures the maintenance of repression when the histones are diluted after replication [43]. The evidence obtained in our study supports the idea that SUUR protein controls the appropriate propagation of histone modifications in the wake of replication fork in Drosophila, thus providing a thought-provoking example of epigenetic inheritance mechanism.

## Methods

### Plasmids

Dam-Pc (KT921801) construct was made using the *hsp70* > *loxP*-*Stop*-*loxP* > *DamMyc* vector (JN993988) containing a stop cassette flanked by lox sites between the *hsp70* minimal promoter and the CDS [39]. Dam-SUUR construct (JN993989) was published in previous study [39]. Complete sequences of the constructs were verified by Sanger sequencing and are available from NCBI (accession numbers are given in parentheses).

### Fly strains

*SuUR* mutation was described in [19] and is available from our laboratory stock. *y* ^*1*^ *w* ^*67c23*^ (further referred to as *y,w*) flies were obtained from the Bloomington Stock Center (# 6599) and used as the wild type control in all experiments. Fly strain for DamID of Pc was generated by inserting the corresponding construct into the attP18 landing site [48]. Flies expressing SUUR-Dam and control Dam-alone were obtained previously [39]. Flies were raised at 23 °C.

### Quantitative PCR

#### Under-replication in polytene chromosomes

Salivary glands (10–20 pairs) were accurately dissected in PBS, fat body was completely removed. Total genomic DNA was isolated using optimized phenol–chloroform extraction method, as detailed below. Salivary glands were quickly homogenized by mixing with 600 µl of lysis buffer (100 mM NaCl, 200 mM Sucrose, 100 mM Tri-HCl pH = 9.1, 50 mM EDTA, 0.5% SDS) and passing the lysate 4–6 times through a 1-ml syringe fitted with 27-gauge needle. 10 µl of Dispase II solution (100 mg/ml) were added and incubated for at least 3 h at 56 °C. Equal volume of phenol–chloroform was added and mixed thoroughly by hand to obtain a uniform emulsion. The mixture was centrifuged at 16,000*g* for 5 min. Upper phase was transferred to a new 1.5-ml tube. Interphase carryover was avoided. Next, 3 µl of RNase A (10 mg/ml) were added to the sample and incubated for 20 min at 37 °C to remove RNA. Equal volume of chloroform was added and mixed thoroughly by hand, followed by centrifugation at 16,000*g* for 5 min to remove RNase A and any traces of phenol. Upper phase was transferred to a new 1.5-ml tube. DNA was precipitated with 1.5 × volume of 100% isopropanol. The pellet was washed thoroughly with 1 ml of 70% ethanol, followed by centrifugation at 16,000*g* for 5 min. The supernatant was removed, and DNA pellet was air-dried for at most 5 min to prevent over-drying. DNA yield was measured using Nanodrop 2000 device. DNA integrity and RNA contamination level were assessed using agarose gel electrophoresis.

DNA polytenization was measured using standard curve method on CFX96 System (Bio-Rad). Genomic DNA from 25 larval ganglia was used to prepare standards, starting from 1 ng/μl concentration, with three successive 5 × dilutions. Genomic DNA from salivary glands was diluted to approximately 0.5 ng/ul. SYBRGreen qPCR reactions were set up using 2 × master mix (Biolabmix, http://biolabmix.ru/). Final concentration of primers in a 25-µl reaction was 0.5 µM. Each reaction contained 10 μl of DNA template. Primers to *Actin 42A* gene were used for the inter-strain normalizations. Each data point was acquired from two biological replicates run in triplicates. The annealing temperature for all primers was 60 °C. The results were analyzed using Bio-Rad CFX software and in MS Excel.

*Actin 42A* primers

**Table:** 

Ac-1	CACGTTTGCTCTGTGCCTCAT
Ac-2	CCGCGTGCAGTTTTTCCTT

The list of primers in the SSRs:

**Table:** 

64C-1	TTCTTCGTCATCGCTTTCTTT
64C-2	CTGGGTGCAGAAGTACAGTGA
71C-1	TTAATCAACTTCAGCGCATTG
71C-2	TGGTTATCAGGTTGTCGTTCA
15D-1	TGACTTCCGTCGCTTTACTG
15D-2	GTCCTGGTCCGTCATCTTTT
30A-1	ACGATCCCAAATGGAAAGAG
30A-2	GTTCCAGCTCCTCAGAGTCC
62C-1	GCTTTGCCATTTGCTGAGTA
62C-2	CCCATGCCATCTCAACTATG
67E-1	CAATGGGTTCTTTGCATTTG
67E-2	CAAGAGGGGTGGTACGTTCT
85A-1	AATGCAATTTCCACGCTTAAC
85A-2	TGCAAAAACAGAAACAGCAAC

The list of primers in pericentric regions

**Table:** 

CG40178-1	TTGCGTTGGTACTGTTCTGG
CG40178-2	GACTCTGGGTGCTTTGCAT
CG17374-1	AGGAGCTAAAACTGCGTGGT
CG17374-2	CCAAACAAACCATCTGAACG
Scp1_F	AATCGTCAGATGAGTTCGTCA
Scp1_R	GAGAGAACGCCAACTCTATATCC
AGO3_F	AAACTCGGTGGAACCAAGAC
AGO3_R	ATGCGAATCTGCAACTCAAC
Gpb5-1	CGTTCCATCCTGTTTGTGTC
Gpb5-2	CTTGCTTACGCTGTCCTCTG

The list of primers in the 25A region

**Table:** 

dp-1	GATACGGATTGTCCCAGTGAA
dp-2	GCAGTACGGTTTTGCAGAGAG
CG15635-1	AACCCCTGGAGTATGGTATCC
CG15635-2	TGGTTTTCCTGAATTGGAAAG
CG3355-1	TCGAGTCAATCAAGACATTCG
CG3355-2	AGCCTGGAAGGGATTTAGAAG
CG15634-1	AGGGTAATCCTCTGGTGAGGT
CG15634-2	AAATTATCATCGAAGGCGAGA
CG15631-1	CCATACCGGAACCCAATAAGT
CG15631-2	GCTCTTCAAAGGACACACAGC
CG3294-1	AAAAACATGCCAAAGAACGTG
CG3294-2	AATTCCTGCATTTCCTCCTGT

The list of primers in the 75C region

**Table:** 

gk-1	TCCAAGAAGCTGATGAAGCTC
gk-2	GGGGTCGCCTACATCCTAATA
CG7320-1	ACTCGATTCGTTCCTGACTGT
CG7320-2	CGGCCAATTTAACAAACTGAT
CheA75a-1	GGTTACCAATGAAAGGTTGGA
CheA75a-2	GTTTTCAGTCCATCGAAGAGC
CG13700-1	GCAACCACTTTAACCACTTC
CG13700-2	ACCACCCATGCCATAGAC
rpr-1	GGGGAAAACCAATAGTCCAGT
rpr-2	GCTGATGAGTGGTGACTGTGT
skl-1	GGTCCTGAAGCAATTTTACCA
skl-2	GTATTTGAACGGTGGCCTTTA
bora-1	GCCTTTCACCCATTAGAAACC
bora-2	TCCAGCTCGTGCATTAGAAGT

### Gene expression analysis

RNA was isolated from 10 to 20 salivary glands with TRIZOL reagent (Invitrogen) according to the manufacturer’s recommendations. 1 µg of total RNA was used in random-primed reverse transcription reaction using VILO kit (Invitrogen). Expression was assessed using standard curve procedure, as described above. *Actin 42A* gene was used for normalizations. Each experiment was repeated twice with three technical replicates each.

**Table:** 

H1-F	AGGCAAAGTCGAAGGTTTTGT
H1-R	TTAGCTTTGGGCTTTTTGTCA
H2A-F	AGTGAAGGGAAAGGCAAAGTC
H2A-R	TTCCATTACGGCAGCTAGGTA
H2B-F	AGGATGGACCTGCTTGAGAAC
H2B-R	AACATCACCAAGACCGACAAG
H3-F	AGTGAAACCCAAATCGGAGAT
H3-R	CGGCCTTAGTAGCCAGTTGTT

### DamID and data analysis

DamID was performed according to the previously published protocol [49, 50]. In brief, we used previously published Drosophila stocks that expressed Dam-SUUR, and Dam-alone proteins under the control of minimal *hsp70* promoter of the pUAST vector [39]. Dam-Pc strain was generated in this study. To avoid position effects, all the constructs were inserted into the same landing site (attP18) on the X-chromosome. Previously, we demonstrated that expression of Dam fusion proteins from transgenes that were integrated at this site is very low and is not detectable by Western blot [39].

The flies were kept at 23 °C. Fifty salivary glands were carefully dissected and fat body was removed completely. DNA was isolated using phenol–chloroform method as described above. 1ug of genomic DNA was digested by DpnI endonuclease that only cuts Dam-methylated GATC sequences. Digested DNA was ligated with double-stranded DNA adapters and then digested with DpnII endonuclease that cuts non-methylated GATC sequences. At this step, adapters remained ligated only to the fragments between two neighboring methylated GATC sequences in the genome. DpnII digestion step is needed to increase the specificity of the method. Next, methylated fragments were selectively amplified using adapter-specific primer. On an agarose gel, DamID products appeared as a smear ranging between 100 and 800 bp.

Library preparation and data analysis were performed as described earlier [36]. Before the preparation of libraries for Illumina DamID-seq, the adapters used in DamID procedure were cut off with DpnII. No further fragmentation was performed. Thus, all the specific DamID fragments would have GATC sequences on both ends. Illumina TruSeq protocol was used for library preparation, followed by sequencing on HiSeq or MiSeq System (pair-end, 50 or 75 bp).

The reads obtained were mapped to BDGP R5/dm3 Drosophila genome assembly using MOSAIK software [51] with the following parameters: -m all -mmp 0.1 -act 20 -a single. For further analysis, only the reads that started with GATC sequence were retained. The number of reads for each genomic fragment between the neighboring GATC sites (GATC fragments) was summed up. Pearson correlation coefficient between two biological replicates was above 0.9. Data filtering, profile generation and FDR-based peak calling were performed exactly as described before [36].

### H3K27me3 ChIP-seq and data analysis

To perform H3K37me3 ChIP-seq, we used the True MicroChIP & MicroPlex Library Preparation™ Package (Diagenode), according to manufacturer’s recommendations. About 20 µl of 0–4-h embryos or 25 pairs of dissected salivary glands were used as starting material. Abcam anti-H3K27me3 antibodies (#6002) were used for chromatin immunoprecipitation throughout the study, except for the specificity tests, where Cell Signaling Technology #9733 anti-H3K27me3 and Millipore #07-452 anti-H3K27me2 antibodies were used (Additional file 2: Figure S1) Formaldehyde-fixed chromatin was sheared using BioRuptor instrument (Diagenode). Three biological replicates were processed. DNA from precipitated material and input were used for library preparation using Illumina Nextera protocol. The libraries were sequenced using MiSeq System (paired end, 75 bp).

The reads were mapped to BDGP R5/dm3 Drosophila genome assembly using MOSAIK software [51] with following parameters: -m all -mmp 0.1 -act 20 -a single. Paired reads were combined. For each genomic position, the RPM (reads per million) value was calculated for ChIP sample and input sample. Log2 of the ChIP to input ratio was used as a measure of local H3K27me3 enrichment. To build the whole-genome profile, the data were smoothed using a 1-kb sliding window, step 100 bp.

To identify the regions enriched with H3K27me3 in wild type strain and depleted for H3K27me3 in *SuUR* mutants, the mhsmm R package (http://www.jstatsoft.org/v39/i04/) was used. Prior to the analysis, the data were quantile normalized. The resulting list of the regions is provided in the Additional files.

## Additional files

**Additional file 1: Table S1.** Coordinates of 193 HMM-defined SSRs. First column—chromosome name, second column—start coordinate, third column—end coordinate. Each line corresponds to a single SSR.

**Additional file 2: Figure S1.** Comparison of ChIP results in *SuUR* mutants and in wild type obtained with H3K27me3 antibodies from different vendors and with the antibodies against H3K27me2. **A**—scatter plot of ChIP-chip signals obtained with the Abcam #6002 antibodies in *SuUR* mutants (abscissa) and in wild type (ordinate) [13]. **B**—scatter plot showing H3K27me3 ChIP-seq signals obtained with Cell Signaling Technology #9733 (CST #9733) antibodies in the same genotypes. **C**—the same analysis performed with Millipore #07-452 antibodies against H3K27me2. Datapoints inside 193 SSRs are shown in red. In both cases (**A** and **B**) H3K27me3 antibodies produce the characteristic skew (arrows): SSRs systematically show stronger signal in wild type strain as compared to *SuUR* mutants. This tendency is absent in case of H3K27me2 (**C**).

**Additional file 3: Table S2.** Coordinates of all reported under-replicated regions [12–14]. The data from three studies were combined in UCSC Table Browser using the UNION function. First column—chromosome name, second column—start coordinate, third column—end coordinate, fourth column—unique ID. Each line corresponds to a single under-replicated region.

**Additional file 4: Figure S2.** Examples of SSRs that are not under-replicated in salivary gland of wild type strain. The color code and legend are the same as in Fig. 1a. H3K27me3 profiles are presented as quantile normalized log_2_(IP/inp) values.

**Additional file 5: Table S3.** Coordinates of 158 HMM-defined SNRs. First column—chromosome name, second column—start coordinate, third column—end coordinate. Each line corresponds to a single SNR.

**Additional file 6: Figure S3.** Comparison of SUUR DamID profiles in Kc167 cells and in salivary gland. Data for Kc167 cells were taken from [5], profile in salivary glands was obtained in this study. The profiles are very consistent, although with some expectable cell type-specific differences (exemplified by black frame).

**Additional file 7: Figure S4.** *SuUR* mutation has no effect of the expression levels of histone genes. Expression of the histone genes was measured using qPCR in *SuUR* mutant salivary glands and in wild type control. No significant difference was detected using *t* test.

## Abbreviations

*SuUR* (SUUR): Suppressor of Under-replication
PCNA: Proliferating cell nuclear antigen
H3K27me3: Histone H3 tri-methylated on lysine 27
H3K9me2/3: Histone H3 di- or tri-methylated on lysine 9
DSB: Double-strand break
HP1: Heterochromatin protein 1
Su(var)3–9: Suppressor of variegation 3–9
PRC: Polycomb-repressive complex
E(z): Enhancer of zeste
Pc: Polycomb
CDS: Coding DNA sequence
DamID: DNA adenine methyltransferase (Dam) identification
HMM: Hidden Markow model
SSR: *SuUR*-sensitive region
SNR: *SuUR*-non-sensitive region
FDR: False discovery rate

## References

[1] Schubeler D, Scalzo D, Kooperberg C, van Steensel B, Delrow J, Groudine M (2002). Genome-wide DNA replication profile for *Drosophila melanogaster*: a link between transcription and replication timing. Nat Genet.

[2] Hiratani I, Takebayashi S, Lu J, Gilbert DM (2009). Replication timing and transcriptional control: beyond cause and effect—part II. Curr Opin Genet Dev.

[3] Woodfine K, Fiegler H, Beare DM, Collins JE, McCann OT, Young BD, Debernardi S, Mott R, Dunham I, Carter NP (2004). Replication timing of the human genome. Hum Mol Genet.

[4] MacAlpine HK, Gordan R, Powell SK, Hartemink AJ, MacAlpine DM (2010). Drosophila ORC localizes to open chromatin and marks sites of cohesin complex loading. Genome Res.

[5] Filion GJ, van Bemmel JG, Braunschweig U, Talhout W, Kind J, Ward LD, Brugman W, de Castro IJ, Kerkhoven RM, Bussemaker HJ (2010). Systematic protein location mapping reveals five principal chromatin types in Drosophila cells. Cell.

[6] Cowell IG, Aucott R, Mahadevaiah SK, Burgoyne PS, Huskisson N, Bongiorni S, Prantera G, Fanti L, Pimpinelli S, Wu R (2002). Heterochromatin, HP1 and methylation at lysine 9 of histone H3 in animals. Chromosoma.

[7] Schwartz YB, Pirrotta V (2007). Polycomb silencing mechanisms and the management of genomic programmes. Nat Rev Genet.

[8] Schotta G, Ebert A, Krauss V, Fischer A, Hoffmann J, Rea S, Jenuwein T, Dorn R, Reuter G (2002). Central role of Drosophila SU(VAR)3–9 in histone H3-K9 methylation and heterochromatic gene silencing. EMBO J.

[9] Danzer JR, Wallrath LL (2004). Mechanisms of HP1-mediated gene silencing in Drosophila. Development.

[10] Andrew DJ, Henderson KD, Seshaiah P (2000). Salivary gland development in *Drosophila melanogaster*. Mech Dev.

[11] Zhimulev IF, Semeshin VF, Kulichkov VA, Belyaeva ES (1982). Intercalary heterochromatin in Drosophila. Chromosoma.

[12] Belyakin SN, Christophides GK, Alekseyenko AA, Kriventseva EV, Belyaeva ES, Nanayev RA, Makunin IV, Kafatos FC, Zhimulev IF (2005). Genomic analysis of Drosophila chromosome underreplication reveals a link between replication control and transcriptional territories. Proc Natl Acad Sci USA.

[13] Sher N, Bell GW, Li S, Nordman J, Eng T, Eaton ML, Macalpine DM, Orr-Weaver TL (2012). Developmental control of gene copy number by repression of replication initiation and fork progression. Genome Res.

[14] Yarosh W, Spradling AC (2014). Incomplete replication generates somatic DNA alterations within Drosophila polytene salivary gland cells. Genes Dev.

[15] Nordman JT, Kozhevnikova EN, Verrijzer CP, Pindyurin AV, Andreyeva EN, Shloma VV, Zhimulev IF, Orr-Weaver TL (2014). DNA copy-number control through inhibition of replication fork progression. Cell Rep.

[16] Lilly MA, Spradling AC (1996). The Drosophila endocycle is controlled by Cyclin E and lacks a checkpoint ensuring S-phase completion. Genes Dev.

[17] Lilly MA, Duronio RJ (2005). New insights into cell cycle control from the Drosophila endocycle. Oncogene.

[18] Andreyeva EN, Kolesnikova TD, Belyaeva ES, Glaser RL, Zhimulev IF (2008). Local DNA underreplication correlates with accumulation of phosphorylated H2Av in the *Drosophila melanogaster* polytene chromosomes. Chromosome Res.

[19] Belyaeva ES, Zhimulev IF, Volkova EI, Alekseyenko AA, Moshkin YM, Koryakov DE (1998). Su(UR)ES: a gene suppressing DNA underreplication in intercalary and pericentric heterochromatin of *Drosophila melanogaster* polytene chromosomes. Proc Natl Acad Sci USA.

[20] Zhimulev IF, Belyaeva ES, Makunin IV, Pirrotta V, Volkova EI, Alekseyenko AA, Andreyeva EN, Makarevich GF, Boldyreva LV, Nanayev RA (2003). Influence of the SuUR gene on intercalary heterochromatin in *Drosophila melanogaster* polytene chromosomes. Chromosoma.

[21] Koryakov DE, Walther M, Ebert A, Lein S, Zhimulev IF, Reuter G (2011). The SUUR protein is involved in binding of SU(VAR)3–9 and methylation of H3K9 and H3K27 in chromosomes of *Drosophila melanogaster*. Chromosome Res.

[22] Pokholkova GV, Koryakov DE, Pindyurin AV, Kozhevnikova EN, Belyakin SN, Andreyenkov OV, Belyaeva ES, Zhimulev IF (2015). Tethering of SUUR and HP1 proteins results in delayed replication of euchromatic regions in *Drosophila melanogaster* polytene chromosomes. Chromosoma.

[23] Kolesnikova TD, Posukh OV, Andreyeva EN, Bebyakina DS, Ivankin AV, Zhimulev IF (2013). Drosophila SUUR protein associates with PCNA and binds chromatin in a cell cycle-dependent manner. Chromosoma.

[24] Andreyeva EN, Bernardo TJ, Kolesnikova TD, Lu X, Yarinich LA, Bartholdy BA, Guo X, Posukh OV, Healton S, Willcockson MA (2017). Regulatory functions and chromatin loading dynamics of linker histone H1 during endoreplication in Drosophila. Genes Dev.

[25] Posukh OV, Maksimov DA, Skvortsova KN, Koryakov DE, Belyakin SN (2015). The effects of SUUR protein suggest its role in repressive chromatin renewal during replication in Drosophila. Nucleus.

[26] Campbell S, Ismail IH, Young LC, Poirier GG, Hendzel MJ (2013). Polycomb repressive complex 2 contributes to DNA double-strand break repair. Cell Cycle.

[27] Johnson DP, Spitz GS, Tharkar S, Quayle SN, Shearstone JR, Jones S, McDowell ME, Wellman H, Tyler JK, Cairns BR (2015). HDAC1,2 inhibition impairs EZH2- and BBAP-mediated DNA repair to overcome chemoresistance in EZH2 gain-of-function mutant diffuse large B-cell lymphoma. Oncotarget.

[28] Belyakin SN, Babenko VN, Maksimov DA, Shloma VV, Kvon EZ, Belyaeva ES, Zhimulev IF (2010). Gene density profile reveals the marking of late replicated domains in the *Drosophila melanogaster* genome. Chromosoma.

[29] Koryakov DE, Pokholkova GV, Maksimov DA, Belyakin SN, Belyaeva ES, Zhimulev IF (2012). Induced transcription results in local changes in chromatin structure, replication timing, and DNA polytenization in a site of intercalary heterochromatin. Chromosoma.

[30] Graveley BR, Brooks AN, Carlson JW, Duff MO, Landolin JM, Yang L, Artieri CG, van Baren MJ, Boley N, Booth BW (2011). The developmental transcriptome of *Drosophila melanogaster*. Nature.

[31] Hammonds AS, Bristow CA, Fisher WW, Weiszmann R, Wu S, Hartenstein V, Kellis M, Yu B, Frise E, Celniker SE (2013). Spatial expression of transcription factors in Drosophila embryonic organ development. Genome Biol.

[32] Tomancak P, Beaton A, Weiszmann R, Kwan E, Shu S, Lewis SE, Richards S, Ashburner M, Hartenstein V, Celniker SE (2002). Systematic determination of patterns of gene expression during Drosophila embryogenesis. Genome Biol.

[33] Tomancak P, Berman BP, Beaton A, Weiszmann R, Kwan E, Hartenstein V, Celniker SE, Rubin GM (2007). Global analysis of patterns of gene expression during Drosophila embryogenesis. Genome Biol.

[34] Fischle W, Wang Y, Jacobs SA, Kim Y, Allis CD, Khorasanizadeh S (2003). Molecular basis for the discrimination of repressive methyl-lysine marks in histone H3 by Polycomb and HP1 chromodomains. Genes Dev.

[35] Makunin IV, Volkova EI, Belyaeva ES, Nabirochkina EN, Pirrotta V, Zhimulev IF (2002). The Drosophila suppressor of underreplication protein binds to late-replicating regions of polytene chromosomes. Genetics.

[36] Maksimov DA, Laktionov PP, Belyakin SN (2016). Data analysis algorithm for DamID-seq profiling of chromatin proteins in *Drosophila melanogaster*. Chromosome Res.

[37] Maksimov DA, Laktionov PP, Posukh OV, Belyakin SN, Koryakov DE (2017). Genome-wide analysis of SU(VAR)3–9 distribution in chromosomes of Drosophila melanogaster. Chromosoma.

[38] Pindyurin AV, Moorman C, de Wit E, Belyakin SN, Belyaeva ES, Christophides GK, Kafatos FC, van Steensel B, Zhimulev IF (2007). SUUR joins separate subsets of PcG, HP1 and B-type lamin targets in Drosophila. J Cell Sci.

[39] Maksimov DA, Koryakov DE, Belyakin SN (2014). Developmental variation of the SUUR protein binding correlates with gene regulation and specific chromatin types in *D. melanogaster*. Chromosoma.

[40] Landais S, D’Alterio C, Jones DL (2014). Persistent replicative stress alters polycomb phenotypes and tissue homeostasis in *Drosophila melanogaster*. Cell Rep.

[41] Lattao R, Bonaccorsi S, Guan X, Wasserman SA, Gatti M (2011). Tubby-tagged balancers for the Drosophila X and second chromosomes. Fly (Austin).

[42] Lee HG, Kahn TG, Simcox A, Schwartz YB, Pirrotta V (2015). Genome-wide activities of Polycomb complexes control pervasive transcription. Genome Res.

[43] Lanzuolo C, Lo Sardo F, Diamantini A, Orlando V (2011). PcG complexes set the stage for epigenetic inheritance of gene silencing in early S phase before replication. PLoS Genet.

[44] Moshkin YM, Alekseyenko AA, Semeshin VF, Spierer A, Spierer P, Makarevich GF, Belyaeva ES, Zhimulev IF (2001). The bithorax complex of *Drosophila melanogaster*: underreplication and morphology in polytene chromosomes. Proc Natl Acad Sci USA.

[45] Nordman JT, Orr-Weaver TL (2015). Understanding replication fork progression, stability, and chromosome fragility by exploiting the suppressor of underreplication protein. BioEssays.

[46] Alabert C, Barth TK, Reveron-Gomez N, Sidoli S, Schmidt A, Jensen ON, Imhof A, Groth A (2015). Two distinct modes for propagation of histone PTMs across the cell cycle. Genes Dev.

[47] Alabert C, Bukowski-Wills JC, Lee SB, Kustatscher G, Nakamura K, de Lima Alves F, Menard P, Mejlvang J, Rappsilber J, Groth A (2014). Nascent chromatin capture proteomics determines chromatin dynamics during DNA replication and identifies unknown fork components. Nat Cell Biol.

[48] Markstein M, Pitsouli C, Villalta C, Celniker SE, Perrimon N (2008). Exploiting position effects and the gypsy retrovirus insulator to engineer precisely expressed transgenes. Nat Genet.

[49] Greil F, Moorman C, van Steensel B (2006). DamID: mapping of in vivo protein-genome interactions using tethered DNA adenine methyltransferase. Methods Enzymol.

[50] Vogel MJ, Peric-Hupkes D, van Steensel B (2007). Detection of in vivo protein–DNA interactions using DamID in mammalian cells. Nat Protocols.

[51] Lee W-P, Stromberg MP, Ward A, Stewart C, Garrison EP, Marth GT (2014). MOSAIK: a hash-based algorithm for accurate next-generation sequencing short-read mapping. PLoS ONE.

