# Supplementary figures and images for: Functional dissection of Drosophila melanogaster SUUR protein influence on H3K27me3 profile
Source: Epigenetics Chromatin. 2017 Dec 1;10:56. doi: 10.1186/s13072-017-0163-z (PMC5709859; doi:10.1186/s13072-017-0163-z)

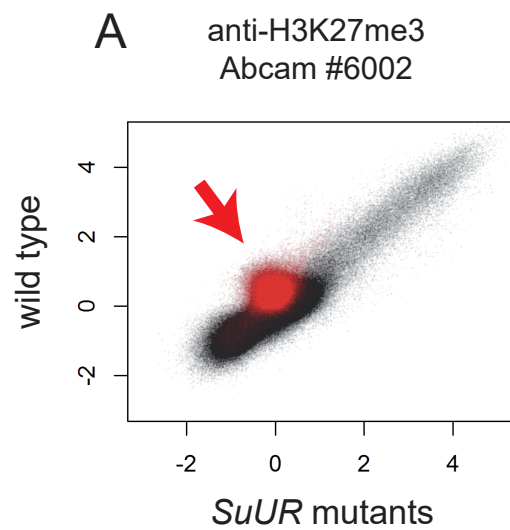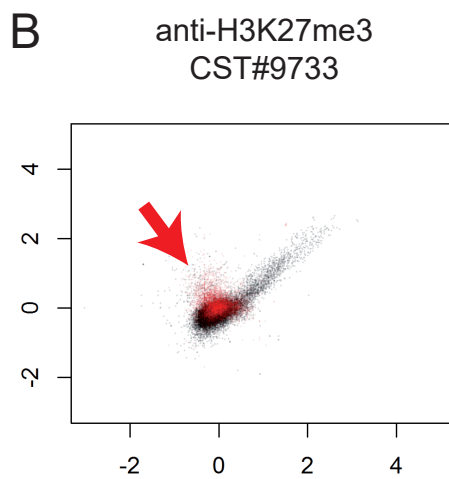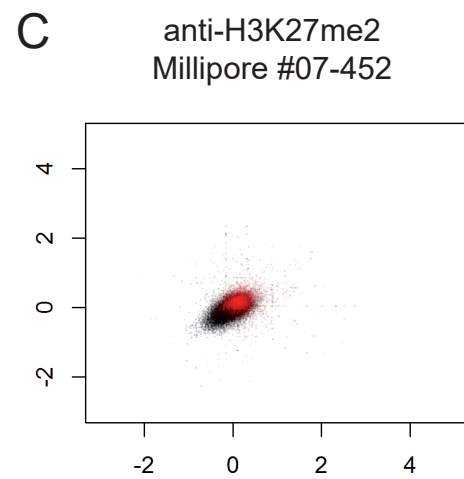

Supplement: Supplementary file 2 — Additional file 2: Figure S1. Comparison of ChIP results in SuUR mutants and in wild type obtained with H3K27me3 antibodies from different vendors and with the antibodies against H3K27me2. A—scatter plot of ChIP-chip signals obtained with the Abcam #6002 antibodies in SuUR mutants (abscissa) and in wild type (ordinate) [13]. B—scatter plot showing H3K27me3 ChIP-seq signals obtained with Cell Signaling Technology #9733 (CST #9733) antibodies in the same genotypes. C—the same analysis performed with Millipore #07-452 antibodies against H3K27me2. Datapoints inside 193 SSRs are shown in red. In both cases (A and B) H3K27me3 antibodies produce the characteristic skew (arrows): SSRs systematically show stronger signal in wild type strain as compared to SuUR mutants. This tendency is absent in case of H3K27me2 (C). [file 13072_2017_163_MOESM2_ESM.pdf]

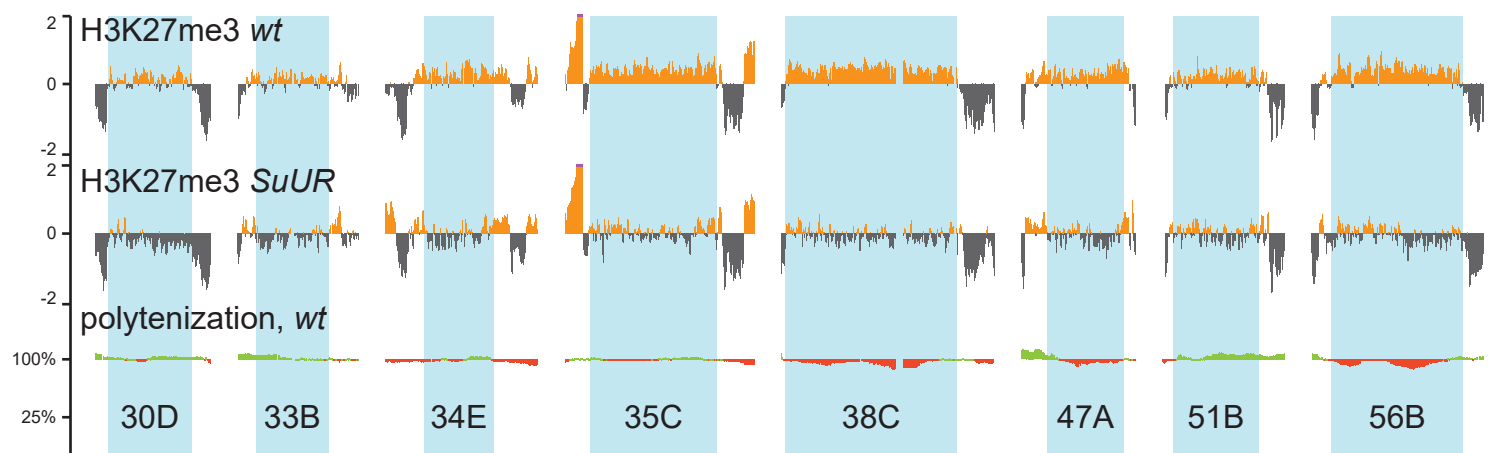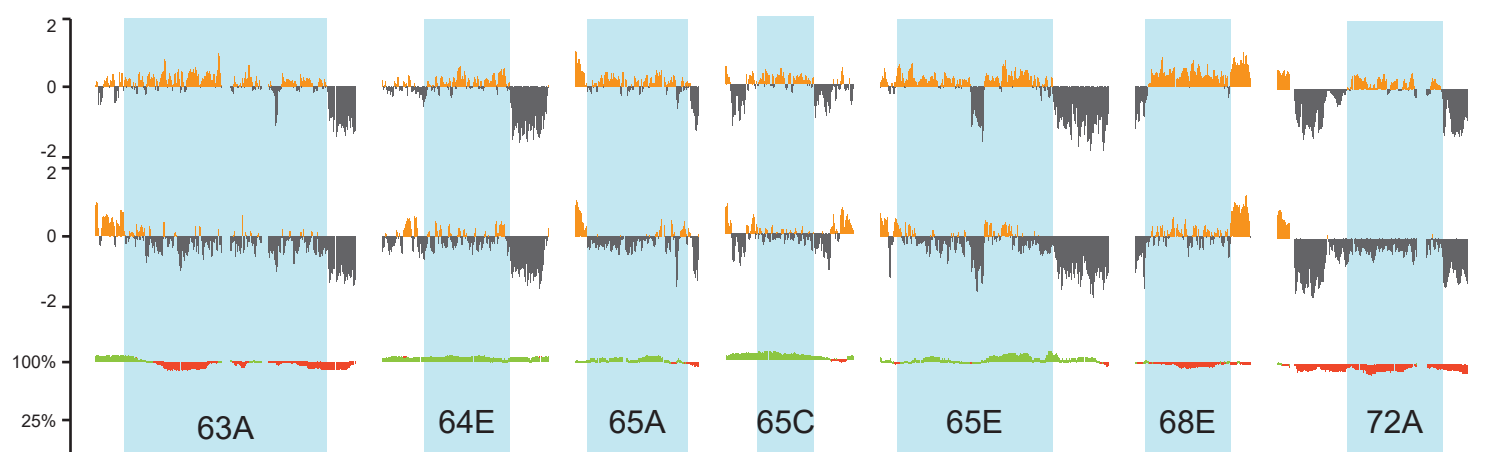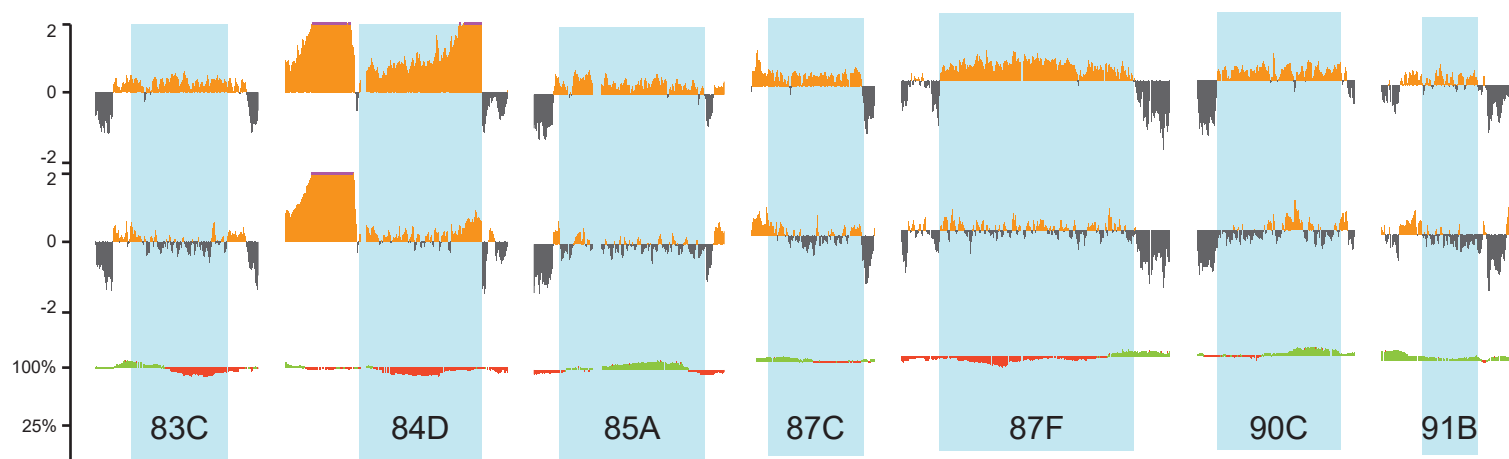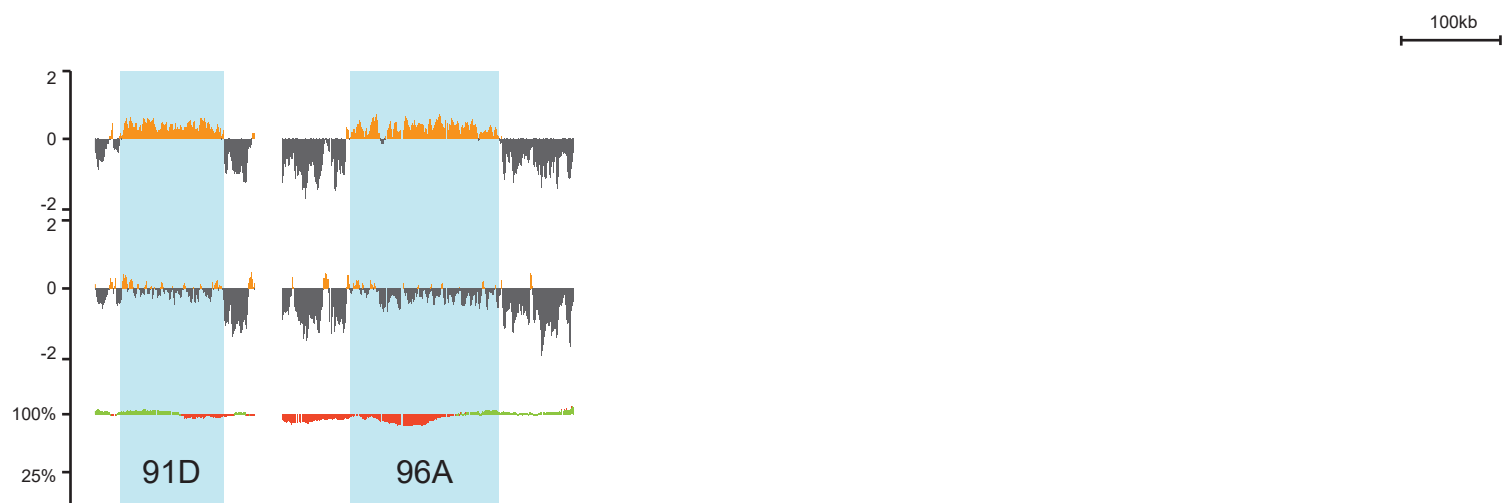

Supplement: Supplementary file 4 — Additional file 4: Figure S2. Examples of SSRs that are not under-replicated in salivary gland of wild type strain. The color code and legend are the same as in Fig. 1a. H3K27me3 profiles are presented as quantile normalized log2(IP/inp) values. [file 13072_2017_163_MOESM4_ESM.pdf]

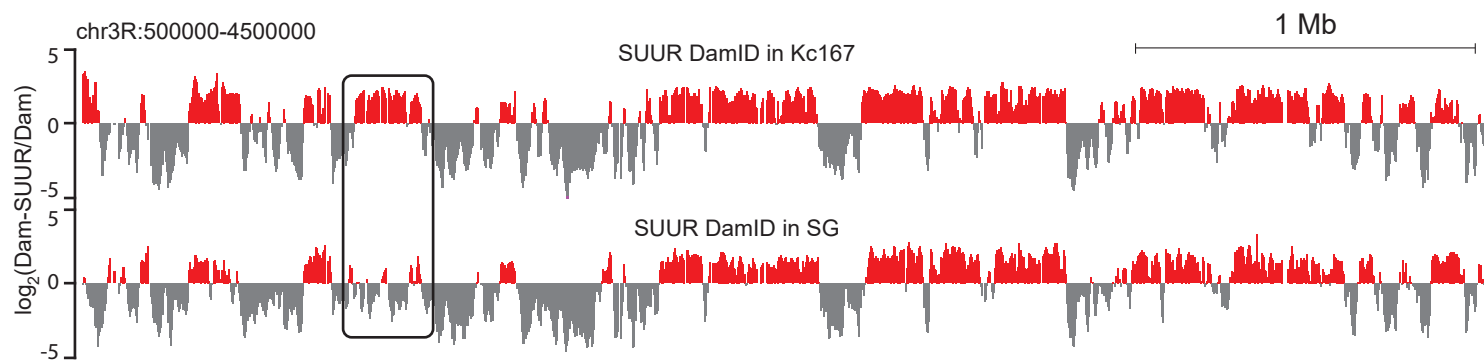

Supplement: Supplementary file 6 — Additional file 6: Figure S3. Comparison of SUUR DamID profiles in Kc167 cells and in salivary gland. Data for Kc167 cells were taken from [5], profile in salivary glands was obtained in this study. The profiles are very consistent, although with some expectable cell type-specific differences (exemplified by black frame). [file 13072_2017_163_MOESM6_ESM.pdf]

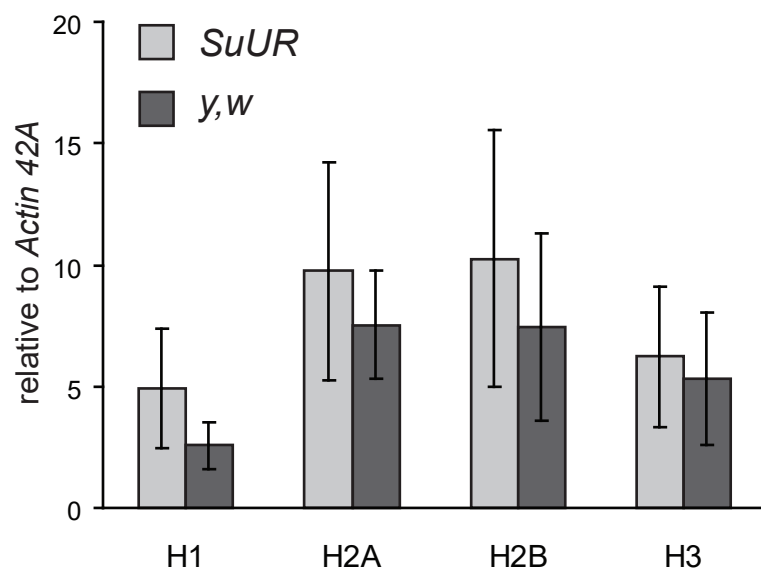

Supplement: Supplementary file 7 — Additional file 7: Figure S4. SuUR mutation has no effect of the expression levels of histone genes. Expression of the histone genes was measured using qPCR in SuUR mutant salivary glands and in wild type control. No significant difference was detected using t test. [file 13072_2017_163_MOESM7_ESM.pdf]
